# Evaluation of feeding ruminal-protected folate and cobalt pectinate on growth performance, carcass characteristics and plasma vitamin B_12_ and folate status in finishing beef steers

**DOI:** 10.1093/tas/txac116

**Published:** 2022-08-24

**Authors:** Alyssa B Word, Ben P Holland, Kendall J Karr, Michael T Socha, Cory Kending, Mark E Branine

**Affiliations:** Cactus Research Ltd., Amarillo, TX 79101, USA; Cactus Research Ltd., Amarillo, TX 79101, USA; Cactus Research Ltd., Amarillo, TX 79101, USA; Zinpro Corporation, Eden Prairie, MN 55344, USA; Zinpro Corporation, Eden Prairie, MN 55344, USA; Zinpro Corporation, Eden Prairie, MN 55344, USA

**Keywords:** feedlot, folate, vitamin B_12_, vitamin B_9_

## Abstract

A large pen feedlot study was conducted to evaluate the response of yearling steers fed novel sources of rumen-protected folate (**RPFA**) and cobalt (cobalt pectinate; **Co-PECT**) on plasma levels of vitamin B_12_ and folate, growth performance, and carcass characteristics. A total of 2,100 steers (initial BW = 381 ± 45.2 kg.) were enrolled in the study at the time of randomization with 2,091 steers started on treatment diets following the transition to the finishing diet. A generalized randomized block design with sampling error (GRBD) with two treatments and 15 pen replications per treatment (5 blocks × 6 pens/block; 30 pens total with 70 steers/pen) were evaluated with pen serving as the experimental unit. A control (**CON**) treatment consisted of the standard finishing diet while the test diet consisted of the standard finishing diet providing 3.0 mg ∙ kg^−1^ DM of RPFA and 1.0 mg ∙ kg^−1^ DM total supplemental cobalt with approximately half coming from Co-PECT (**TEST**). Blood samples were collected from 60 randomly selected steers at study initiation and prior to shipping for plasma B_12_ and folate measurement. Data were analyzed with the model including fixed effects of treatment, block, and treatment within block interaction. Live growth performance was not affected by treatment; however, carcass-adjusted performance and hot carcass weight were numerically improved by TEST in 3 of the 5 blocks (treatment × within block interaction, *P* ≤ 0.03) of cattle. Plasma levels for both folic acid and vitamin B_12_ were extremely low at study initiation and increased over the course of the feeding period. Feeding TEST increased (*P* < 0.01) plasma B_12_ levels compared to CON by the completion of the trial; however, mean levels would still be considered marginal. Plasma folate was lower (*P* < 0.05) in TEST steers at the beginning of the study, with no difference between treatments by the time cattle were shipped. Results suggested that cattle coming into the feedlot may be of low or marginal status in both plasma folate and vitamin B_12_. While the status of folate and B_12_ improved in both CON and TEST with days on feed, providing RPFA and Co-PECT further helped improve vitamin B_12_ status; although, overall levels remained low, which may have affected the overall response to RPFA. Additional research is required to better understand the role of B vitamin supplementation for growing-finishing feedlots and develop methods for assessing the status and improving potential responses.

## INTRODUCTION

Currently, no requirements for folate or other B vitamins have been established for beef cattle (NASEM, 2016) where the prevailing dogma has been the endogenous microbial synthesis of B-vitamins has been adequate to meet the needs of the animal (Zinn et al., 1987; Schwab et al., 2006). However, modern dairy and beef cattle are genetically capable of markedly greater production than their predecessors. In addition, the application of growth-enhancing technologies such as anabolic implants and beta-agonists will increase the physiological capability for enhanced muscle accretion and may increase requirements for folate and other micro-nutrients in excess of amounts that can be synthesized endogenously or absorbed from conventional dietary sources. Little research has evaluated folate in modern growing-finishing feedlot cattle, although research in lactating dairy cows has indicated the benefits of folate supplementation (Girard et al., 1995; Girard and Matte, 1998; Graulet et al., 2007). A novel ruminal-protected source of folate (**RFPA)** evaluated by Deters et al. (2021) indicated linear increases in plasma folic acid (**FA**) at the end of the growing and finishing phases and decreased plasma glucose after the completion of the growing phase. Results from this study also indicated several other responses in growth, DM intake, and reductions in the percentage of liver abscesses that suggested a physiological response in growing-finishing cattle fed RPFA. Therefore, the objective of this study was to evaluate the same RPFA along with a novel source of cobalt (cobalt pectinate; **Co-PECT**) on growth performance, carcass characteristics, and plasma levels of folate and vitamin B_12_ in yearling steers fed in a commercial research feedlot.

## MATERIALS AND METHODS

### Test Animals

All experimental procedures in this study complied with FASS (2020) guidelines and Cactus Research standard procedures for animal handling and welfare and were under the supervision of the staff veterinarian. Between 13 August 2019 and 5 October 2019 2,441 primarily black-hided, English and English × Continental crossbred yearling steers (mean BW 360 kg) were purchased from sources in Texas, Oklahoma, Alabama, Mississippi, and Tennessee and shipped to the study site in the Texas panhandle. At initial processing steers were vaccinated against viral respiratory pathogens (Bovi-Shield Gold^®^ IBR-BVD; Zoetis, Florham Park, NJ); administered parasiticide to control internal and external parasites (Synanthic^®^; Boehringer Ingelheim Vetmedica, Duluth, GA, and Dectomax^®^; Zoetis,) and implanted with Revalor-XS^®^ (Merck Animal Health, Summit, NJ). A computer-generated randomization schedule was used to assign candidate animals to study pens within blocks. Within each group of six candidate animals in the chute at processing, one was randomly assigned to each study pen in the block. Within blocks, study pens were then randomly assigned to treatments. Cattle were blocked by the time of arrival at the feedlot with each block (five blocks total) having six pens (70 steers/pen) with three pen replications for each dietary treatment. A total of 2,100 steers (initial BW = 381 ± 45.2 kg.) were enrolled in the study at the time of randomization. Study initiation (day 0), for all blocks, was defined as the first-day treatment diets were fed and occurred after the transition to the finishing diet was completed at which time 2,091 steers were weighed and started on treatment diets. Blocks 1 and 2 were processed on-arrival (14 August 2019–15 August 2019 and were returned to pens to complete the transition to finish diet after which time they were then returned to the processing barn and were randomized to treatments on 24 September 2019 and 26 September 2019, respectively with study day 0 occurring on 30 September 2019, the first-day treatment diets were fed. Blocks 3–5 were processed and were randomized to treatments at the same time and were transitioned to finish diets within their treatment pens before study day 0 occurred. Enrolled steers were individually identified with duplicate color-coded ear tags which denoted a unique six-digit number that identified the lot number and the individual animal within the lot. Ear tags were colored so that adjacent pens had differently colored tags.

Steers were group weighed by pen using a platform scale on study day 0 and the morning of shipment to the packing plant for the calculation of live growth performance. Body weights (**BW**) were measured prior to the morning feeding and a 2% pencil shrink was applied to day 0 BW and a 4% shrink was applied to final BW. Steers were observed daily for abnormalities and unusual observations were recorded by exception. Animals exhibiting signs of bovine respiratory disease (**BRD**), digestive disturbance, lameness, injury, or other malady were taken to a hospital facility where further evaluation and treatment, if necessary, were performed according to the standard treatment protocol. Animals evaluated as not growing at the same rate as pen-mates, non-responsive to BRD treatment, or injured and buller animals were removed from the experiment with the date, body weight and reason for removal and BW recorded. Animals that died during the experiment had a field necropsy performed and the date of death and diagnosis, when determinable, were recorded.

### Feeding Management and Treatment Diets

Steers were housed in soil-surfaced, outdoor pens measuring 53.4 m deep and 18.3 m wide with an 18.3 m feed bunk and 3.1 m concrete bunk apron. Pens were equipped with a fence-line constant-flow concrete water tank and equally stocked to provide approximately 14 m^2^/steer and 25 cm/steer of linear bunk space. Feed bunks were visually checked daily at approximately 0600 h, 1800 h, and 2100 h for the presence of residual feed with the 0600-h evaluation serving as the primary check. Feed calls were made to provide feed to appetite such that feed carry-over in the bunk was minimized. In addition, large increases or decreases in daily feed calls were avoided if possible. The starter diet for all treatments was Ramp^®^ (Cargill Corn Milling, Bovina, TX). Loose grass hay was top-dressed on the feed bunk for at least 3 days after arrival. Transition to the finishing diet was conducted using a two-ration system where systematically 10–15% of starting diet was substituted with an equal quantity of the finishing diet. Increases in the amount of finishing diet were made every 2–4 d. Pens were fed 3 times daily using a truck-mounted with a Roto-Mix^™^ (Dodge City, KS) delivery box and with daily amounts delivered recorded electronically to the nearest 4.5 kg. A common basal finishing diet was prepared in the on-site feed mill with the primary ingredients consisting of steam-flaked corn, Sweet Bran Plus^®^ (Cargill Corn Milling, Bovina, TX), wet distiller’s grains plus solubles, and other ingredients common to the High Plains cattle feeding region (Table 1). Test articles and other micro-ingredients were dispensed with water via a micro-ingredient machine (Micro Technologies, Amarillo, TX) directly onto the mixer truck and allowed to mix on the truck for approximately 5 min. The typical batch size was approximately 3,600 kg. Monensin (Rumensin^®^ Elanco Animal Health, Indianapolis, IN) was included in the starting diet and finishing diets at 22 and 46.9 mg ∙ kg^−1^ DM, respectively. Tylosin (Tylan^®^, Elanco Animal Health, Indianapolis, IN) was included in the finish diet at 8.25 mg ∙ kg^−1^ DM. During the 29–33 days prior to slaughter all steers were fed ractopamine HCl (**RAC**; Optaflexx^®^; Elanco Animal Health) at 30.0 mg ∙ kg^−1^ ∙ DM. Basal diet targeted total dietary Zn from supplemental sources at approximately 120 mg Zn ∙ kg^−1^ DM, achieved by including 30 mg Zn ∙ kg^−1^ DM from Propath^™^ Zn (Zinpro Corporation, Eden Prairie, MN) and 30 mg Zn ∙ kg^−1^ DM from Availa^™^ Zn 120 (Zinpro Corporation) with the remainder (60 mg Zn ∙ kg^−1^ DM) of the supplemental Zn provided by an inorganic source, zinc sulfate. A malfunction in the micro-ingredient machine caused the ProPath Zn product not to be dispensed into the ration for both dietary treatments for an estimated 12 days beginning approximately 26 December 2019. The problem was discovered and resolved on 07 January 2020.

**Table 1.** Formulated ingredient composition of finishing diet (DM basis)

Item ^2^	CON ^1^	TEST ^1^
Steam-flaked corn, %	54.63	54.63
Sweet bran plus, %	18.34	18.34
Wet distiller’s grains, %	17.75	17.75
Corn stalks or cotton burrs, %	7.46	7.46
Glycerin, %	0.44	0.44
Yellow grease, %	1.33	1.33
Micro-ingredients	0.05	0.05
Vitamin A, IU	545	545
Vitamin D	54.5	54.5
Monensin, mg ∙ kg^−1^	47	47
Tylosin, mg ∙ kg^−1^	8.25	8.25
Zn from Availa Zn 120, mg ∙ kg^−1^	30.0	30.0
Zn from Propath Zn 170, mg ∙ kg^−1^	30.0	30.0
Zn from zinc sulfate, mg ∙ kg^−1^	60.0	60.0
Folate from RPF, mg ∙ kg^−1^	–	3.0
Cobalt from inorganic source, mg ∙ kg^−1^	0.55	0.55
Co from cobalt pectinate, mg ∙ kg^−1^	–	0.50

CON = standard diet providing 120 mg ∙ kg^−1^ Zn from Availa Zn, Propath Zn and ZnSO_4_; TEST = standard diet including 3.0 mg ∙ kg^−1^ RPFA and 0.50 mg ∙ kg^−1^ Co from cobalt pectinate.

Weighted average formulation of treatment diets.

Treatments evaluated were: 1. Control (**CON**): The standard finishing diet used at the study site. 2. Experimental premix (**TEST**) was added to the standard finishing diet providing 3.0 mg ∙ kg^−1^ DM of RPFA and 0.50 mg ∙ kg^−1^ DM Co-PECT to provide a total dietary level of 1.0 mg Co ∙ kg^−1^ DM.

### Sample Collection and Analytical Procedures

Samples of each diet were collected daily from the feed bunk and subsamples were dried at 100°C. Daily dry matters were averaged weekly and used for the calculation of dry matter intake (**DMI**). An additional sample from each diet was collected monthly and submitted to a commercial laboratory for nutrient analysis. The nutrient composition of experimental diets is reported in Table 2. At study initiation of blocks 1 and 4, 15 steers from each treatment were randomly selected for collection of a blood sample by jugular venipuncture. The same steers (*n* = 14 or 13 for CON in Block 1 and Block 4, respectively; *n* = 15 for TEST for both Blocks 1 and 4) were removed from their pens and a second blood sample was collected (142 or 110 days on treatments for Block 1 and 4, respectively; 9 or 48 days from harvest for Blocks 1 and 4, respectively). Plasma was isolated and shipped to Applied Biosciences (College Station, TX) for analysis of B_12_ and folic acid concentrations using radioimmunoassay. Clinical serum or plasma values for assessing the adequacy or deficiency of either vitamin B_12_ or FA in beef cattle are minimal; therefore, criteria for assessing the status of the serum folate and vitamin B_12_ levels measured in this study were based on recommendations from the analytical laboratory as well as from previously published research. Herdt and Hoff (2011) suggested an optimal serum concentration of vitamin B_12_ in cattle was 200 to 400 pg/mL. Other research has suggested levels > 400 pg/mL as adequate, 200–400 pg/mL as marginal, and < 200 pg/mL as a clinical (functional) deficiency (Fisher and MacPherson, 1990). Based on this information and for this research, vitamin B_12_ and folate concentrations were classified into three categories where B_12_ concentrations < 150 pg/mL were below the detection limit of the laboratory. Values of 150–200 pg/mL were considered marginal and values > 200 pg/mL considered adequate. Similarly, for plasma folate, levels < 11 ng/mL were classified as low; values between 11 and 15 ng/mL classified as adequate; and values > 15 ng/mL classified in excess.

**Table 2.** Mean values for the analyzed composition of finishing diets^1, 2^

	Sample set 1^3^		Sample set 2^3^	
Item	CON	TEST	CON	TEST
Dry matter, %	57.4 ± 1.6	56.9 ± 1.6	60.2 ± 3.2	60.2 ± 3.2
Crude protein, %	15.9 ± 0.6	16.0 ± 0.6	15.5 ± 0.7	15.5 ± 0.7
Acid detergent fiber, %	12.3 ± 0.3	12.9 ± 0.6	8.8 ± 4.8	8.8 ± 4.8
Neutral detergent fiber, %	22.2 ± 1.1	23.2 ± 1.0	21.3 ± 0.4	21.3 ± 0.4
Crude fat, %	5.6 ± 0.4	5.8 ± 0.02	5.5 ± 0.5	5.5 ± 0.5
Calcium, %	0.82 ± 0.09	0.77 ± 0.08	0.86 ± 0.06	0.86 ± 0.06
Phosphorus, %	0.55 ± 0.03	0.55 ± 0.03	0.52 ± 0.05	0.52 ± 0.05
Magnesium, %	0.26 ± 0.02	0.27 ± 0.01	0.24 ± 0.01	0.24 ± 0.01
Potassium, %	0.86 ± 0.02	0.87 ± 0.03	0.84 ± 0.05	0.84 ± 0.05
Sulfur, %	0.26 ± 0.01	0.26 ± 0.01	0.23 ± 0.01	0.23 ± 0.01
Sodium, %	0.23 ± 0.05	0.23 ± 0.05	0.19 ± 0.04	0.19 ± 0.05
Cobalt, mg/kg	1.71 ± 0.16	2.38 ± 0.21	0.25 ± 0.15	0.66 ± 0.14
Copper, mg/kg	17.0 ± 0.82	16.8 ± 1.0	14.4 ± 1.14	14.8 ± 1.30
Iron, mg/kg	262.5 ± 93.3	279.5 ± 99.6	184.6 ± 90.4	180.4 ± 55.84
Manganese, mg/kg	62.3 ± 5.74	64.3 ± 6.4	56.8 ± 4.0	56.0 ± 3.39
Zinc, mg/kg	143.5 ± 16.8	150.3 ± 15.7	135.8 ± 19.1	130.2 ± 20.49

CON = standard diet providing 120 mg ∙ kg^−1^ Zn from Availa Zn, Propath Zn and ZnSO_4_; TEST = standard diet including 3.0 mg ∙ kg^−1^ RPFA and 0.50 mg ∙ kg^−1^ Co from cobalt pectinate.

Weighted averages from analysis of treatment diets with values derived from four samples collected from 30 September to 24 November, 2019 (Sample Set 1) and five samples collected from 25 November 2019 to 14 April 2020 (Sample Set 2).

Sample sets 1 and 2 were collected before and after the trace mineral correction, respectively.

### Carcass Measurements

Cattle in each pen had projected days on feed assigned by the Cactus Feeders cattle marketing projection program. Prior to RAC feeding, pens of cattle were visually evaluated for condition and BW. Pens within a block were shipped on the same day to Tyson Fresh Meats (Amarillo, TX) for slaughter and processing. For blocks 1 and 2 trained personnel from the Beef Carcass Research Center (**BCRC**; West Texas A&M University, Canyon, TX) recorded individual animal ear tag numbers to document the kill sequence and affixed a harvest sequence number to each carcass. Plant carcass ID and hot carcass weight (**HCW**) were recorded and verified by carcass sequence number. Livers were scored for the presence and severity of abscesses using a standard system based on the number and severity of visual abscesses as well as the presence of other abnormalities. For blocks 3, 4, and 5, carcass data were pen averages due to policies related to COVID-19 which prevented the collection of individual animal data. Carcass data analyzed for these blocks were lot averages of HCW, quality and yield grade distributions, as well as camera data reported by the plant that included marbling score (**MARB**), backfat thickness (**BFAT**), and ribeye area (**REA**). Liver scores were only available from carcasses in the first two blocks shipped. Carcasses were graded after approximately 36 h chill by USDA graders for the quality grade (**QG**) while yield grade (**YG**) was assigned by the in-house camera system. The dressing percentage for each pen was calculated as the mean hot carcass weight/mean shrunk live weight × 100. Empty body fat (**EBF**) was calculated according to Guiroy et al. (2002).

### Statistical Analysis

Statistical analyses were conducted using SAS 9.4 (SAS Inc., Cary, NC). The experimental design was a generalized randomized block design with sampling error (GRBD) where sampling error allowed measurement of the variation among the three replicates of the same treatment within the same block. There were 15 pen replications (5 blocks × 6 pens/block; 30 pens total with 70 steers/pen) per treatment. Data were analyzed using pen as the experimental unit. Growth performance was calculated on a “deads and removals-excluded” basis. Carcass weight gain was calculated assuming an initial 58% dressing percentage when steers arrived at the feedlot. Categorical data (removals, mortality, carcass grade distributions, and liver scores) were analyzed using logistic regression. Because initial BW was different (*P* = 0.02) between treatments, initial BW (*P* < 0.001) was included as a covariate in subsequent data analyses. Plasma B_12_ and folate status were analyzed as both continuous as well as categorical data. For analysis as continuous data, Proc Univariate (SAS 9.4) was used initially to evaluate the normality of the data. Data were then analyzed using Proc MIXED with sampling time as repeated measures to assess the effects of treatment, block, treatment*block interaction, and sampling time. Analyses of categorical data were conducted using Pearson’s chi-squared test in Stata 16 for each of the two time-points to evaluate the degree of association between plasma status and treatment. Significance was defined as *P* ≤ 0.10 and a trend was defined as 0. 10 < *P* ≤ 0.15.

## RESULTS

### Dietary Analysis

Because cobalt is used by rumen microbes to synthesize vitamin B_12_ and to ensure a vitamin B_12_ deficiency did not influence the response to RPFA, it was supplemented to all steers. In the basal diet, 0.55 mg Co/kg DM from an inorganic source was added. In the TEST diet, an additional 0.55 mg Co/kg DM from Co-PECT was included as a supplemental source of Co. Cobalt pectinate is currently being evaluated as a more bioavailable source of Co for rumen bacteria. Laboratory analyses of diet samples prior to 25 November 2019, indicated average Co concentration was 1.71 mg/kg DM or 2.38 mg/kg DM for CON and TEST, respectively. Following correction of the trace mineral package, the average Co concentration was 0.25 or 0.66 mg/kg, respectively for CON and TEST diet samples. In both CON and TEST diets, the level of Co provided was substantially above the recommended Co requirement for beef cattle of 0.15 mg Co/kg DM, (Tiffany et al., 2003; NASEM, 2016) although, greatly below the maximum tolerable concentration of 25 mg/kg DM.

### Blood Analysis

The number of steers based on status category for analyzed values of plasma B_12_ and folic acid are reported in Table 3 and Table 4, respectively. Plasma B_12_ status was not different (*P* = 0.96) at study initiation, although only 6 of 57 (10.5%) animals tested were considered adequate with 39 of 57 (68.4%) undetectable, while 12 of 57 (21.1%) were of marginal status. The proportion of steers in each category was also not different (*P* = 0.42) between dietary treatments when measured prior to shipping. Only 1 of 57 (1.8%) samples collected at the end of the feeding period was undetectable while 27 (47.4%) were marginal and 29 (50.9%) were adequate, indicating a general increase in plasma B_12_ status during the feeding period. Mean B_12_ values (Table 5) were increased (*P* ≤ 0.01) in TEST steers prior to shipping by approximately 26% compared to CON. The number of steers with a low FA status at processing tended (*P* = 0.13) to be greater in those animals assigned to TEST compared to CON treatment. Across both treatments, 4 of 57 (7.0%) steers sampled were within the adequate category at study initiation while 41 (71.9%) were low and 12 (21.1%) were in excess. Samples collected towards the end of the feeding period indicated the number of animals within each category of FA status was not different (*P* = 0.35) between dietary treatments. Across treatments, the number of steers within the adequate category increased with 22 of 57 steers (38.6%), 20 steers (35.1%) low, and 15 steers (26.3%) in excess. Similar to the B_12_ status, these data indicated a relative improvement in plasma FA status during the feeding period. Mean FA levels at study initiation were lower (*P* ≤ 0.05) in TEST steers compared to CON; however, samples collected prior to shipping indicated similar folate levels between treatments.

**Table 3.** Summary of animals based on plasma B_12_ status at study initiation and prior to shipment ^1^

	Study initiation^3^		Prior to shipment^2, 3^	
Category	CON	TEST	CON	TEST
No. animals per category				
Undetectable (<150 pg/mL) ^4^	18	21	1	0
Marginal (150–200 pg/mL)	6	6	14	13
Adequate (>200 pg/mL)	3	3	12	17
Total	27	30	27	30
Mean, maximum and minimum values				
Mean (pg/mL)	52.81	55.93	193.15	240.7
Minimum (pg/mL)	12.00	1.00	1.00	151.0
Maximum (pg/mL)	255.00	211.00	271.00	464.0

CON = standard diet providing 120 mg ∙ kg^−1^ Zn from Availa Zn, Propath Zn, and ZnSO_4_; TEST = standard diet including 3.0 mg ∙ kg^−1^ RPFA and 0.50 mg ∙ kg^−1^ Co from cobalt pectinate.

Plasma collection prior to shipment occurred on the same day for two different blocks such that 29 steers had received treatment diets for 142 days and 28 animals had received treatment diets for 110 days.

*P* values for samples collected on day 0 or prior to shipment were 0.96 and 0.42, respectively as based on Chi-square statistic.

Values that were reported as undetectable (<150 pg/mL) were assigned as 1 for calculation of mean.

**Table 4.** Summary of animals based on plasma folate status at study initiation and prior to shipment^1^

	Study initiation^3^		Prior to shipment^2,3^	
Category	CON	TEST	CON	TEST
No. animals per category				
Low (<11 ng/mL)	16	25	10	10
Normal (11–15 ng/mL)	3	1	8	14
Above normal (>15 ng/mL)	*8*	*4*	*9*	*6*
Total	27	30	27	30
Mean, maximum and minimum values				
Mean, ng/mL	10.5	6.4	14.6	13.5
Minimum, ng/mL	3.2	0.88	7.2	7.6
Maximum, ng/mL	20.7	22.2	26.0	23.7

CON = standard diet providing 120 mg ∙ kg^−1^ Zn from Availa Zn, Propath Zn, and ZnSO_4;_ TEST = standard diet including 3.0 mg ∙ kg^−1^ RPFA and 0.50 mg ∙ kg^−1^ Co from cobalt pectinate.

Plasma collection prior to shipment occurred on the same day for two different blocks such that 29 steers had received treatment diets for 142 days and 28 animals had received treatment diets for 110 days.

*P* values for samples collected on arrival or prior to shipment were 0.13 and 0.35, respectively as based on Chi-square statistic.

**Table 5.** Effects of feeding RPFA and cobalt pectinate on plasma B_12_ and folate levels of yearling steers

	Treatment ^1^			*P*-values ^3^	
Item	CON	TEST	SEM ^2^	Treatment	Covariate^4^
No. samples, *n*^5^	27	30			
Plasma B_12_, pg/mL					
Study initiation	58.9	55.9	13.9	0.88	
Prior to shipping	191.2	240.7	13.3	<0.01	
Plasma folate, ng/mL					
Study initiation	10.40	8.36	0.75	0.05	
Prior to shipping ^d^	14.09	13.93	0.88	0.90	<0.01

CON = standard diet providing 120 ppm Zn from Availa Zn, Propath Zn, and ZnSO_4_; TEST = standard diet including 3.0 ppm RPFA and 0.50 mg ∙ kg^−1^ Co from cobalt pectinate with 27 samples for CON and 30 samples for TEST at arrival and pre-shipping, respectively.

Standard error of least squares means, with highest value reported.

*P*-values based on *F* statistic.

Plasma values for folate at study initiation were used as a covariate.

Plasma collection prior to shipment occurred on the same day for two different blocks such that 29 steers had received treatment diets for 142 days and 28 animals had received treatment diets for 110 days.

### Animal Removal and Mortality

Removals and mortalities are reported in Table 6. There was no difference (*P* ≥ 0.34) in the number of steers dying between treatments during the time treatment diets were being fed. The cause of death was evenly distributed among the various categories listed. The overall mortality rate was 0.81% which would be acceptable for yearling steers fed at the test facility. Likewise, the number of animals requiring removal from the study was similar between treatments (*P* ≤ 0.99) with an overall mean removal of 0.67% of the animals fed treatment diets until shipping. The number of animals requiring medical attention throughout the study that remained on the study was minimal.

**Table 6.** Animal accountability, mortality, and removals of cattle fed RPFA and cobalt pectinate.

	Treatment ^1^		
Item	CON	TEST	*P*-value
No. allotted to study, *n*	1,050	1,050	–
Prior to initiation of treatments			
No dying, *n*	4	1	–
No removed, *n*	2	2	–
No. starting study, *n*	1,044	1,047	–
No dying during trial, *n*			
Digestive, *n*	3	3	–
Respiratory, *n*	1	3	–
Other ^2^*, n*	2	*5*	–
Total, *n* (%)	6(0.57)	11 (1.05)	0.34
No removed during trial, *n*			
Buller, *n*	1	0	–
Digestive, *n*	0	1	–
Other, *n*	4	5	–
Respiratory*, n*	*1*	*2*	–
Total, *n* (%)	12 (0.57)	16 (0.76)	0.99
No shipped, *n*	1,032	1,028	–
No. carcasses, *n*	1,032	1,025	–

CON = standard diet providing 120 mg ∙ kg^−1^ Zn from Availa Zn, Propath Zn, and ZnSO_4_; TEST = standard diet including 3.0 mg ∙ kg^−1^ RPFA and 0.50 mg ∙ kg^−1^ Co from cobalt pectinate.

Diagnosis of “other” includes peritonitis, lameness or injury, urinary calculi, or traumatic reticuloperitonitis.

### Feedlot Performance

Initial BW was greater (*P* = 0.02; Table 7) for CON compared to TEST therefore, this value was used as a covariate in all subsequent analyses of growth performance and carcass measurements. Dry matter intake was not different (*P* = 0.28) between treatments during the study period. Final BW, total weight gain, average daily gain (**ADG**), and feed efficiency (**DMI:ADG** or **ADG:DMI**) were likewise not different between dietary treatments (*P* ≥ 0.42).

**Table 7.** Effects of feeding RPFA and cobalt pectinate on body weight and growth performance of yearling steers—live animal basis

	Treatment ^1^			*P*-values ^3^	
Item	CON	TEST	SEM ^2^	Treatment	Covariate ^4^
Initial BW, kg	410	407	0.82	0.02	–
DM intake, kg ∙ hd^−1^ ∙ d^−1^	9.87	9.71	0.17	0.28	0.46
Dead and Removals excluded					
Days on feed, d	158	158	–	–	**–**
Final BW, kg	642	642	1.3	0.91	<0.01
Total mean gain, kg	234	234	1.3	0.88	0.87
Daily gain, kg	1.48	1.48	0.01	0.91	0.79
DM feed: gain, kg	6.69	6.60	0.12	0.42	0.56
Gain: DM feed, kg	0.149	0.152	0.003	0.32	0.46

CON = standard diet providing 120 ppm Zn from Availa Zn, Propath Zn and ZnSO_4;_: TEST = standard diet including 3.0 ppm RPFA and 0.50 mg ∙ kg^-1^ Co from cobalt pectinate.

Standard error of least squares means, *n* = 15.

*P*-values based on *F* statistic.

Initial BW used as covariate.

### Carcass Measurements

Carcass-adjusted growth performance and other carcass measurements are shown in Table 8. Carcass-adjusted final BW and ADG were not different between CON and TEST (*P* ≥ 0.26); although, carcass-adjusted feed efficiency tended (*P* ≤ 0.14) to be improved for TEST. Hot carcass weight was not different (*P* = 0.24) between dietary treatments. The dressing percentage was improved (*P* ≤ 0.07) in TEST compared to CON; however, no treatment effects were evident for MARB, REA, BFAT, or EBF. A block × treatment interaction (*P* ≤ 0.04) was observed for HCW, carcass-adjusted values for final BW, total gain ADG, and daily carcass gain (Table 9) with greater values for TEST in blocks 2, 3, and 5, but less than CON in block 1 and similar to CON in block 4. Percentage distribution of USDA quality and yield grades (Table 10) were not different (*P* ≥ 0.18) between dietary treatments, with the exception of a tendency (*P* = 0.10) for an increased percentage of YG 3 carcasses in TEST compared to CON. Liver scores were recorded only during the harvest of blocks 1 and 2 (Table 10). There were no differences between treatments for either the percentage of non-abscessed livers (*P* = 0.40) or livers with other pathology (*P* = 0.54). The percentage of livers with any degree of abscess tended to be reduced by approximately 8.3% in TEST compared to CON (*P* = 0.12).

**Table 10.** Effects of feeding RPFA and cobalt pectinate on percentage distribution of liver abscesses, USDA quality and yield grades

	Treatment ^1^			P-values^3^
Item	CON	TEST	SEM^2^	Treatment
No. carcasses, *n*	1,032	1,025		
USDA quality grade, % of carcasses				
Prime carcasses	3.52	2.62	0.64	0.77
Choice carcasses	75.59	72.14	1.41	0.18
Select carcasses	19.97	23.63	1.34	0.29
No roll carcasses	0.58	0.68	0.63	0.99
USDA yield grade, % of carcasses				
YG 1 carcasses	5.52	8.69	0.90	0.30
YG 2 carcasses	33.41	33.39	0.38	0.38
YG 3 carcasses	41.18	42.47	1.55	0.10
YG 4 carcasses	16.79	12.66	1.18	0.87
YG 5 carcasses	1.81	1.33	0.004	0.45
Heavy carcasses ^4^, % of carcasses	14.3	12.7	0.42	0.33
Liver abscesses, % of livers^6^				
No. livers scored	415	417		
Normal	73.74	74.66	2.16	0.40
Abscessed	15.59	14.29	1.79	0.12
Other defects^5^	10.06	10.54	1.50	0.54

CON = standard diet providing 120 ppm Zn from Availa Zn, Propath Zn, and ZnSO_4_; TEST = standard diet including 3.0 ppm RPFA and 0.50 mg ∙ kg^−1^ Co from cobalt pectinate.

Standard error of least squares means, *n* = 15.15.615.

*P*-values based on *F* statistic.

Carcasses > 454 kg.

Other defects include cirrhosis, contamination, flukes, and telangiectasia.

Liver scores were assessed and recorded on individual animals in blocks 1 and 2 by Beef Carcass Research Center personnel.

**Table 8.** Effects of feeding RPFA and cobalt pectinate on carcass adjusted performance and other carcass measurements of yearling steers

	Treatment ^1^			*P*-values ^3^	
Item	CON	TEST	SEM ^2^	Treatment	Covariate ^4^
No. carcasses, *n*	1,032	1,025			
Carcass adjusted performance					
Final BW, kg^8^	641	643	1.5	0.28	< 0.01
Total mean gain, kg^8^	233	235	1.5	0.27	0.16
Daily gain, kg^8^	1.47	1.48	0.01	0.27	0.14
DM feed: gain, kg	6.74	6.58	0.10	0.14	0.18
Gain: DM feed, kg	0.149	0.153	0.002	0.11	0.13
Initial carcass wt., kg^5^	237	237	0.04	0.97	< 0.01
Total carcass gain, kg^8^	174	176	1.0	0.26	0.10
Other carcass measurements					
Hot carcass wt. kg^8^	411	413	2.8	0.24	<0.01
Dressing percentage, %	64.00	64.30	0.01	0.05	0.04
Ribeye area, cm^2^	93.87	94.13	0.58	0.77	0.71
Marbling score^6^	482	481	5.6	0.89	0.08
Backfat, cm	1.57	1.52	0.03	0.34	0.74
Empty body fat, %^7^	31.14	30.92	0.21	0.49	0.43

CON = standard diet providing 120 ppm Zn from Availa Zn, Propath Zn and ZnSO_4_; TEST = standard diet including 3.0 ppm RPFA and 0.50 mg ∙ kg^−1^ Co from cobalt pectinate.

Standard error of least squares means, *n* = 15.

*P*-values based on *F* statistic.

Initial BW used as covariate.

Calculated using an initial dressing percentage of 58% at study day 0.

Marbling score: 300–399 = slight; 400–499 = small; 500–599 = modest.

Empty body fat calculated according to Guiroy et al. (2002).

Treatment × block interaction, *P* ≤ 0.05.

**Table 9.** Effects of feeding RPFA and cobalt pectinate on treatment × within block interactions for carcass adjusted performance and hot carcass weight^1^

	Block 1		Block 2		Block 3		Block 4		Block 5	
Item	CON	TEST	CON	TEST	CON	TEST	CON	TEST	CON	TEST
Final BW, kg	609	601	598	608	661	667	673	672	662	668
Total gain, kg	201	200	190	200	253	259	265	264	254	259
Daily gain, kg	1.31	1.25	1.26	1.32	1.51	1.55	1.68	1.67	1.63	1.64
Carcass gain, kg	154	149	147	153	187	191	195	194	188	191
HCW, kg	391	386	383	390	425	428	432	431	425	429

Treatment means within a block resulting from significant treatment * within block interaction.

## DISCUSSION

Relatively little published research is available regarding B-vitamin supplementation in feedlot cattle. Earlier work feeding vitamin supplements, including supplementing multiple B vitamins to newly received, stressed calves coming into the feedlot have reported a range of responses. Cole et al. (1979) and Zinn et al (1987) observed reductions in apparent morbidity while Lee et al (1985) indicated improvements in rate and efficiency of gain by a combination of vitamin E and a mixture of B-vitamins during a 28-d starting period. The variability in response to B vitamin supplementation may likely be influenced by the relative amounts of endogenous microbial synthesis and degradation occurring concurrently within the rumen. Previous research has indicated only 3–5% of dietary folic acid escapes microbial degradation within the rumen (Zinn et al, 1987; Santschi et al., 2005). However, despite the challenges in defining a specific requirement for FA, numerous studies have indicated a need for supplemental FA in ruminant diets based on age, breed, stage of production, and other criteria (Girard et al., 1989, 2005; Dumoulin et al., 1991; Preynat et al., 2009).

As suggested by Deters et al. (2021) the observed physiological or production responses to supplemental FA have generally relied on feeding large quantities to compensate for ruminal degradation or by parenteral administration such as weekly injections. Because FA is absorbed primarily in the proximal duodenum (Girard and Rémond, 2002) the animal requires an adequate post-ruminal supply to meet requirements for maintenance, reproduction, and growth. Hence, developing methods to protect dietary FA or other B vitamins from ruminal breakdown may have merit as a means to improve and optimize production efficiency. The target RPFA dose in this study was 3.0 mg RPFA ∙ kg^−1^ DM based on the results of a dose–response study reported by Deters et al. (2021) indicating this as the lowest dietary level which exhibited a positive response in carcass weight relative to the control. In previous work (Zinpro unpublished) a 10% increase in plasma FA concentrations was measured when sheep were orally supplemented with unprotected FA. When sheep were fed RPFA plasma FA was increased approximately by 150%. Assuming only 3% FA is undegraded in the rumen and 100% of the FA not degraded in the rumen is absorbed, the metabolizable value for the folic acid provided by the RPFA was approximately 45%

Results for plasma FA and B_12_ status indicated a majority of animals sampled at study initiation would be considered to be deficient or marginal in status. In the current study, FA and B_12_ concentrations at the beginning of the study averaged 10.4 ng/mL and 58.9 pg/mL, respectively, with the mean FA level being considered low and the mean B_12_ level reflecting the number of animals with B_12_ levels below the analytical detection level that were arbitrarily assigned a value of 1 to facilitate analysis of the data. Deters et al. (2021) reported similar FA values in the calves at study initiation of approximately 12 ng/mL; while B_12_ levels were not reported since 90% of the study animals had undetectable levels. Duplessis et al., (2020) reported on a cross-sectional study of 22 dairy herds in the United States, where plasma folate concentrations averaged 13.4 ng/mL in 427 cows without FA supplementation. Similar information on beef cattle is minimal. In a survey of cattle in different phases of production, Dubeski et al. (1992) reported average plasma B_12_ concentration of 160 pg/mL in 14 feedlot cattle tested (range 97–297 pg/mL), and an average of 251 pg/mL in 13 stressed beef calves (range 116–396 pg/mL). The average of a feedlot animal in that report would have been near the limit of detection in the current trial. Dubeski et al. (1992) also reported a mean plasma FA concentration of 26.0 ng/mL in 14 feedlot cattle tested (range 14.2–35.2 ng/mL), and of 9.7 ng/mL in 13 stressed calves (range 6.4–14.6 ng/mL) indicating FA concentration observed at study initiation in the present study was similar to values reported by Dubeski (1992) for stressed calves. Vitamin B_12_ values prior to shipping in the present study were similar to those reported by Deters et al. (2021) for CON steers (191.5 vs. 196 pg/mL, respectively).

In both the current study and Deters et al. (2021) significant increases in B_12_ status and blood levels were observed even for cattle that were not provided supplemental RPFA. In the current trial, feeding RPFA and an additional source of Co significantly increased B_12_ concentration above CON (241. 2 vs. 191.6 pg/mL, respectively); however, these values may be considered borderline deficient or marginally adequate at best given the criteria utilized in this research. For plasma FA, linear increases were observed by Deters et al. (2021) by feeding RPFA at increasing levels of 3.0, 6.0, 9.0, 12.0, and 15.0 mg/kg DM in samples collected prior to shipping to the packing plant. In the current study, a difference in plasma FA prior to shipping was observed; however, mean plasma FA increased by 3.9 and 5.3 ng/mL for CON and TEST, respectively during the course of the 156-d feeding period. The relative increase in plasma FA in both treatments may have also been facilitated by the extended feeding of the finishing diet were feeding higher concentrate diets has been associated with increased concentrations of folate in the rumen (Hayes et al., 1966; Girard et al., 1994) and plasma (Duplessis et al., 2020).

In addition to the responses discussed above regarding the effects of supplemental RPFA on plasma levels of FA and B_12_, in the present study and the results reported by Deters et al. (2021), other similarities in response between the two studies may be noted. Primarily, while Deters et al (2021) did not indicate a significant polynomial dose response to supplemental RPFA, a comparison between the control diet and that providing 3.0 mg RPFA/kg DM did exhibit a 6 kg increase in HCW. As observed in this study, the treatment × block interaction resulted in three of the five blocks having a numerical advantage in HCW as well. Likewise, Deters et al. (2021) observed steers supplemented with RPFA at 30 or 60 mg/d tended to have a greater percentage of livers with no abscesses (93.4%) compared to steers receiving no supplemental RPFA (79.3%). In the current trial, while liver abscess incidence was greater, feeding RPFA at 30 mg/d resulted in a tendency for a reduction in the percentage of livers with abscesses. In both the present study and Deters et al. (2021) more consistent responses in HCW and other production parameters may have been complicated by the interrelationship between FA and vitamin B_12_ and variation among individual animals regarding their respective status and level of FA and B_12_ adequacy.

Because of the novelty of the RPFA evaluated and in the absence of other corroborating information making definitive conclusions regarding the potential efficacy of providing a rumen-protected source of FA or other B vitamins is not currently feasible. Other studies where a rumen-protected source of FA (Li et al., 2016; Wang et al., 2016) or a rumen-protected blend of B vitamins have been fed (Evans and Mair, 2013; Kaur et al., 2018) have reported positive responses. Previous research as well as the more recent information provided by Deters et al. (2021) in addition to the present research would indicate blood levels of both FA and vitamin B_12_ may be inadequate at critical times to meet demands for reproduction, lactation, growth, or health in dairy and beef cattle. In a recent review, González-Montaña et al. (2020) cited several studies (Girard, and Matte, 2005; Graulet et al., 2007; Preynat et al., 2009; Duplessis et al., 2014, 2017; Brito et al., 2015) that concluded parenteral supplementation of vitamin B_12_ and folate to dairy cows in early lactation improved the efficiency of energy metabolism perhaps by increasing gluconeogenesis. Whether similar improvements in energy metabolism could be made in beef cattle through dietary supplementation of rumen-protected forms of these vitamins remains open to further investigation. Likewise, further research evaluating the RPFA used in this study is required to better define an optimal dose and to evaluate potential responses in feedlot cattle including stressed calves being received into the feedlot.
